# Coarse-to-Fine Contrast Maximization for Energy-Efficient Motion Estimation in Edge-Deployed Event-Based SLAM

**DOI:** 10.3390/mi17020176

**Published:** 2026-01-28

**Authors:** Kyeongpil Min, Jongin Choi, Woojoo Lee

**Affiliations:** Chung-Ang University, 84, Heukseok-ro, Dongjak-gu, Seoul 06974, Republic of Korea; yj070204@cau.ac.kr (K.M.); nex1248@cau.ac.kr (J.C.)

**Keywords:** event-based vision sensor, visual simultaneous localization and mapping (visual SLAM), motion estimation, contrast maximization, coarse-to-fine optimization, edge computing, FPGA prototyping, low-power design

## Abstract

Event-based vision sensors offer microsecond temporal resolution and low power consumption, making them attractive for edge robotics and simultaneous localization and mapping (SLAM). Contrast maximization (CMAX) is a widely used direct geometric framework for rotational ego-motion estimation that aligns events by warping them and maximizing the spatial contrast of the resulting image of warped events (IWE). However, conventional CMAX is computationally inefficient because it repeatedly processes the full event set and a full-resolution IWE at every optimization iteration, including late-stage refinement, incurring both event-domain and image-domain costs. We propose coarse-to-fine contrast maximization (CCMAX), a computation-aware CMAX variant that aligns computational fidelity with the optimizer’s coarse-to-fine convergence behavior. CCMAX progressively increases IWE resolution across stages and applies coarse-grid event subsampling to remove spatially redundant events in early stages, while retaining a final full-resolution refinement. On standard event-camera benchmarks with IMU ground truth, CCMAX achieves accuracy comparable to a full-resolution baseline while reducing floating-point operations (FLOPs) by up to 42%. Energy measurements on a custom RISC-V–based edge SoC further show up to 87% lower energy consumption for the iterative CMAX pipeline. These results demonstrate an energy-efficient motion-estimation front-end suitable for real-time edge SLAM on resource- and power-constrained platforms.

## 1. Introduction

Visual simultaneous localization and mapping (visual SLAM) is a core capability for intelligent platforms such as mobile robots, autonomous vehicles, and drones, enabling concurrent ego-localization and environment reconstruction [1,2,3]. Although a SLAM pipeline typically consists of multiple modules (e.g., feature extraction, data association, mapping, and optimization), the motion estimation module in the front-end plays a particularly critical role, as it directly affects tracking robustness, map quality, and the long-term drift behavior of the entire system [1,2,4,5]. Conventional motion estimation has been largely developed around frame-based RGB cameras, which assume periodic sampling and dense image processing. This assumption often leads to high computational load and power consumption, making it increasingly problematic in resource-limited edge settings where real-time operation and energy efficiency must be simultaneously satisfied [6,7]. In addition, frame-based cameras are susceptible to motion blur under fast dynamics and may suffer from degraded performance in low-light environments or under extreme illumination changes due to noise amplification and exposure limitations.

Event-based vision sensors, commonly referred to as Dynamic Vision Sensors (DVSs) [8], have emerged as a compelling alternative sensing modality for motion estimation under such challenging conditions [7,9,10,11,12,13,14]. Instead of transmitting full image frames, a DVS asynchronously outputs events only when per-pixel brightness changes exceed a threshold. This sensing principle provides (i) microsecond-level temporal resolution, (ii) inherent immunity to motion blur, (iii) a wide dynamic range robust to abrupt illumination variations, and (iv) sparse, scene-dependent data generation that naturally suppresses redundant processing [15,16,17]. These characteristics make event cameras well aligned with the requirements of high-speed robotics and energy-constrained edge perception, motivating extensive research on event-based motion estimation.

Existing event-based motion estimation approaches have diverged into multiple directions. On one hand, learning-based methods have been introduced to improve estimation accuracy in complex scenarios [18,19,20,21,22]. On the other hand, sensor fusion strategies combine events with inertial measurements [23,24,25] or with standard intensity images [26,27,28] to enhance robustness and generalization. While effective in many cases, these approaches often introduce practical limitations for edge deployment, including reliance on large-scale training data, synchronization and calibration overhead across sensing modalities, and increased system complexity. As a result, motion estimation methods with predictable computational structure and strong energy efficiency remain highly desirable for edge-centric robotics pipelines [17].

**Figure 1:**
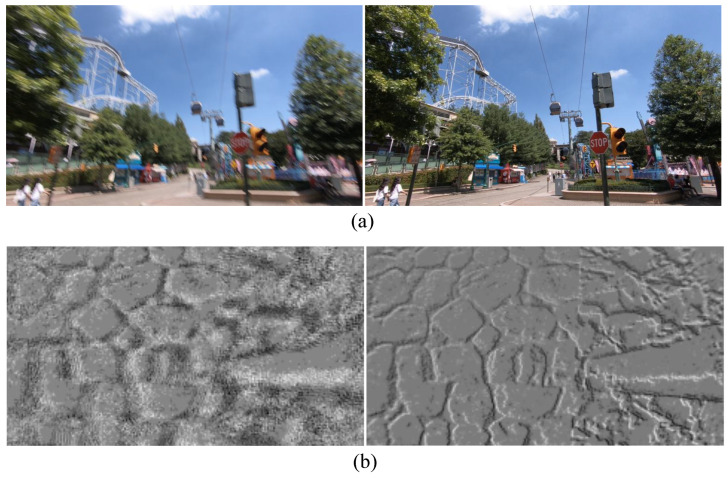
Qualitative comparison between frame-based and event-based motion compensation. (**a**) Frame-based (RGB) vision, where motion compensation aims to align image intensities across frames (REDS dataset [33]). (**b**) Event-based vision, where correct ego-motion alignment causes events generated by the same scene edges to spatially accumulate, resulting in a high-contrast image of warped events (IWE) (Event Camera Dataset [34]).

In this context, learning-free and sensor-fusion-free geometric approaches that rely solely on event streams provide an attractive foundation for edge deployment. Among them, contrast maximization (CMAX) has become a widely adopted framework for estimating motion by directly maximizing the spatial contrast of motion-compensated events [10,11]. Given a motion hypothesis, events are temporally warped to a common reference time, accumulated into an image of warped events (IWE), and evaluated using a contrast objective. When the motion hypothesis matches the true ego-motion, events generated by the same physical edges become spatially aligned, resulting in a sharper and higher-contrast IWE; otherwise, the IWE appears blurred due to misalignment, as illustrated in Figure 1. Owing to this direct geometric interpretation, CMAX has served as a key building block across a broad range of event-based vision problems, including rotational motion estimation [10,13,29], feature-flow estimation [23,30], and motion segmentation [14,31,32].

Despite its effectiveness, conventional CMAX is inherently iterative and computationally demanding. Each optimization iteration repeatedly performs (i) per-event warping and accumulation, typically implemented via bilinear voting, and (ii) image-domain operations such as smoothing and contrast or gradient evaluation. Accordingly, the computational burden is dominated by two terms: an event-domain cost that scales with the number of warped events *N*, and an image-domain cost that scales with the IWE resolution H×W, where *H* and *W* denote the height and width of the IWE grid. With warm-start initialization and gradient-based iterative optimization, CMAX typically exhibits a coarse-to-fine convergence behavior: early iterations capture dominant motion components through large parameter updates, whereas later iterations focus on fine refinement with diminishing returns. However, standard implementations apply the same computational granularity across all iterations, incurring nearly identical cost even when the marginal benefit of refinement becomes limited. This mismatch between optimization needs and computational effort is particularly inefficient on edge platforms, where energy budget, memory bandwidth, and execution predictability are tightly constrained.

To address this issue, we propose ***CCMAX** (coarse-to-fine contrast maximization)* for rotational ego-motion estimation. This paper focuses on contrast maximization-based rotational angular velocity estimation performed through iterative optimization in the SLAM front-end, rather than addressing the entire event-based SLAM pipeline. CCMAX explicitly aligns computation with the coarse-to-fine nature of contrast maximization by introducing two complementary strategies: (i) **coarse-to-fine IWE construction**, which progressively increases the IWE resolution across optimization stages to reduce image-domain cost in early iterations; and (ii) **coarse-grid event subsampling**, which removes redundant event contributions within coarse spatial bins to reduce event-domain cost during coarse stages. By allocating higher computational fidelity only when fine refinement is required, CCMAX systematically reduces the dominant cost of the CMAX pipeline while preserving estimation accuracy. We evaluate CCMAX on standard event-camera benchmarks using IMU-based ground truth and demonstrate that carefully designed coarse-to-fine schedules achieve accuracy comparable to a full-resolution CMAX baseline with substantially reduced computation. Furthermore, our analysis shows that CCMAX reduces floating-point operations (FLOPs) by up to 42%, and energy measurements on an FPGA-based prototype edge SoC report up to 87% energy reduction for the iterative CMAX pipeline under coarse configurations.

The remainder of this paper is organized as follows. Section 2 reviews the principles of contrast maximization and analyzes its computational structure in edge settings. Section 3 introduces CCMAX, detailing coarse-to-fine IWE construction and coarse-grid event subsampling. Section 4 evaluates the proposed approach in terms of accuracy, floating-point operations (FLOPs), and energy efficiency on an edge prototype platform. Section 5 discusses limitations of the proposed approach and outlines directions for future work. Finally, Section 6 concludes the paper.

## 2. Contrast Maximization: Principles and Edge-Oriented Analysis

Following the motivation in Section 1, we review the CMAX framework for learning-free event-based ego-motion estimation and then analyze its computational structure from an edge-deployment perspective. We focus on rotational ego-motion estimation, which is one of the most widely adopted settings of CMAX and directly matches the target problem addressed in Section 3.

### 2.1. Rotational Ego-Motion Estimation via Contrast Maximization

**Event representation.** An event camera outputs asynchronous events when the change in log-intensity at a pixel exceeds a contrast threshold. Each event is represented as

(1) $$e_{k}=\left(x_{k},y_{k},t_{k},p_{k}\right),$$

where $\left(x_{k},y_{k}\right)$∈$R^{2}$ denotes the image-plane location, $t_{k}$ the timestamp, and $p_{k}\in \left\{+1,-1\right\}$ the polarity (brightness increase or decrease). We consider a short temporal segment $\left[t_{0},t_{0}+\Delta t\right]$ and define the associated event window as $E={\left\{e_{k}\right\}}_{k=1}^{N}$, where *N* is the number of events in the window. CMAX estimates ego-motion by finding the motion parameters that best align events in this window.

**Geometric interpretation of event warping.** Under the pure-rotation assumption, a scene point projects to an image bearing that rotates according to the camera angular velocity $\omega \in R^{3}$. Let $\tilde{x}$ denote a homogeneous image coordinate (projective ray) corresponding to an image point x. Assuming constant angular velocity within the short window, the bearing evolves as

(2) $$\tilde{x}\left(t\right)∼R\left(t\right)\;{\tilde{x}}_{0},\;R\left(t\right)=\exp\left(\hat{\omega }\;t\right),$$

where $\left(\hat{\cdot }\right)$ maps a vector to a skew-symmetric matrix and ∼ denotes equality up to scale in homogeneous coordinates. This constant angular velocity assumption is standard in window-based contrast maximization frameworks and enables tractable motion compensation over short temporal intervals [10]. Accordingly, an event $e_{k}$ occurring at time $t_{k}$ can be warped to the reference time $t_{0}$ as

(3) $$x_{k}^{\prime }=W\left(x_{k};\omega ,t_{k}-t_{0}\right),$$

so that all events are compared in a common time frame. For short windows and small inter-event rotation, a first-order approximation is commonly used [10]:

(4) $$x_{k}^{\prime }\approx x_{k}+\left(t_{k}-t_{0}\right)\;\left(\omega \times x_{k}\right),$$

which reduces computational overhead and enables efficient gradient computation in practice, making it well-suited for iterative contrast maximization in event-based rotational motion estimation.

**Image of Warped Events (IWE).** Given a motion hypothesis ω, warped events are accumulated to form an IWE,

(5) $$I\left(x;\omega \right)=\sum_{k=1}^{N}p_{k}\;\delta \;\left(x-x_{k}^{\prime }\left(\omega \right)\right),$$

where δ(·) is the Dirac delta. Directly working with δ is not practical in discrete implementations and, moreover, optimization requires differentiating the objective with respect to ω. Therefore, standard CMAX implementations approximate event accumulation on a pixel grid using *bilinear voting* (i.e., distributing a sub-pixel event contribution to the four neighboring pixels) [10]. This discretization also enables stable numerical gradients by approximating spatial derivatives on the pixel grid via finite differences.

Because the IWE is inherently sparse and can yield a non-smooth objective surface, CMAX typically optimizes a smoothed IWE, obtained by convolving *I* with a small Gaussian kernel (e.g., σ=1 pixel) [10]:

(6) $$I_{\sigma }\left(x;\omega \right)=\left(I\left(x;\omega \right)*G_{\sigma }\right)\left(x\right).$$

This smoothing spreads each event contribution locally, improves the continuity of the contrast functional, and facilitates convergence of gradient-based optimization.

**Figure 2:**
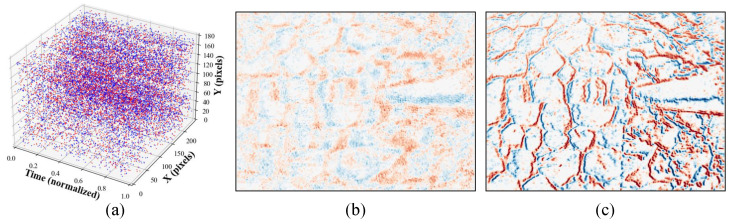
Effect of motion hypothesis on event alignment in contrast maximization. (**a**) Raw events within an event window. (**b**) IWE formed under an inconsistent motion hypothesis ω, where events remain spatially dispersed, resulting in low contrast. (**c**) IWE formed under the consistent hypothesis $\omega^{*}$, where events originating from the same scene edges align spatially, producing a sharper and higher-contrast IWE.

**Contrast maximization and intuition.** Events are predominantly triggered by moving intensity edges; events originating from the same physical edge form coherent spatiotemporal trajectories. If ω matches the true motion, warping brings these trajectories into spatial agreement, causing events from the same physical edges to spatially accumulate and reinforce edge structures in $I_{\sigma }$, as illustrated in Figure 2. CMAX quantifies this alignment by maximizing the spatial contrast of $I_{\sigma }$. In this work, we define contrast as the variance of $I_{\sigma }$ over the valid image domain Ω:

(7) $$C\left(\omega \right)=\mathrm{Var}\;\left(I_{\sigma }\left(x;\omega \right)\right)=\frac{1}{\left|\Omega \right|}\int_{\Omega }{\left(I_{\sigma }\left(x;\omega \right)-\mu \right)}^{2}\;dx,$$

where |Ω| is the area (or, in discrete form, the number of pixels) and $\mu ≜\frac{1}{\left|\Omega \right|}\int_{\Omega }I_{\sigma }\left(x;\omega \right)\;dx$ is the spatial mean. In typical scenes, positive/negative polarities are often roughly balanced and μ can be close to zero, so maximizing C(ω) encourages the IWE to become spatially *peaky*, which corresponds to strong edge alignment.

**Gradient of the contrast functional.** Efficient numerical optimization requires the gradient of C(ω). For the variance-based contrast, the gradient can be expressed as

(8) $$\frac{\partial }{\partial \omega }C\left(\omega \right)=\frac{2}{\left|\Omega \right|}\int_{\Omega }\left(I_{\sigma }\left(x;\omega \right)-\mu \right)\frac{\partial I_{\sigma }\left(x;\omega \right)}{\partial \omega }\;dx.$$

Since $I_{\sigma }=I*G_{\sigma }$, the derivative commutes with convolution, yielding

(9) $$\frac{\partial I_{\sigma }\left(x;\omega \right)}{\partial \omega }=\left(\frac{\partial I(x;\omega )}{\partial \omega }\right)*G_{\sigma }\left(x\right).$$

In practice, ∂I/∂ω is obtained alongside event warping by differentiating the discretized (bilinear-voted) accumulation with respect to the motion parameters, and spatial derivatives are approximated by finite differences on the pixel grid.

**Optimization and warm-up strategy.** Rotational ego-motion estimation is formulated as

(10) $$\omega^{*}=\arg\max_{\omega }C\left(\omega \right).$$

Because C(ω) is generally non-convex, CMAX employs iterative gradient-based optimization. Classic choices include conjugate-gradient methods such as Fletcher–Reeves (CG–FR) [35] or Polak–Ribière variants [36], which have been shown to produce comparable solutions in common event-motion settings [10].

For streaming operation, the event stream is partitioned into consecutive event windows $\left\{E_{m}\right\}$. After solving (10) for window $E_{m}$, the estimate ${\hat{\omega }}_{m}$ is used to initialize the next window $E_{m+1}$ (warm-start), leveraging the fact that angular velocity varies slowly over short time intervals [10]. This assumption is widely adopted in event-based rotational motion estimation, as short event windows are typically used to ensure that angular velocity can be reasonably approximated as locally constant. This warm-start strategy improves convergence speed but does not change the per-iteration computational structure of the CMAX pipeline, which we analyze next.

### 2.2. Edge-Oriented Cost Decomposition of CMAX

**Figure 3:**
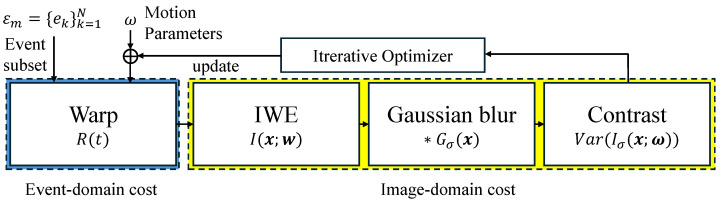
Conventional CMAX computation pipeline repeated at each optimization iteration. Given a motion hypothesis, events are warped and accumulated into an IWE, followed by contrast and gradient evaluation to update the motion parameters. Blue and yellow blocks highlight event-domain and image-domain costs, respectively, both of which are incurred with fixed granularity at every iteration.

Figure 3 summarizes the conventional CMAX computation loop. Given a motion hypothesis ω, each iteration evaluates the same fixed pipeline: (i) warp events to the reference time, (ii) accumulate them into an IWE, and (iii) compute contrast and its gradient to update ω via an iterative optimizer.

From an edge-deployment perspective, the per-iteration cost is dominated by two terms. First, the **event-domain cost** scales with the number of processed events *N*. Event warping, coordinate transforms, and bilinear voting are performed per event; thus both arithmetic operations and memory accesses grow linearly with *N*. Second, the **image-domain cost** scales with the IWE resolution H×W. Gaussian smoothing, contrast computation (variance), and gradient-related image operations traverse the full pixel grid, so their cost grows linearly with the number of pixels. As a result, each iteration incurs approximately O(N) event-domain work and O(HW) image-domain work, and the total cost per window scales linearly with the iteration budget.

**Figure 4:**
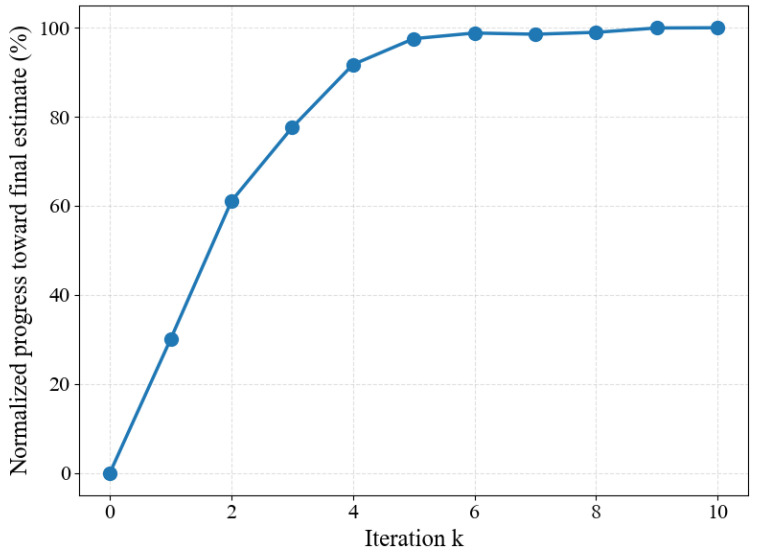
Empirically observed coarse-to-fine convergence behavior of CMAX. Early optimization iterations yield large parameter updates that capture dominant motion components, while later iterations mainly perform fine refinement with diminishing improvements.

A key observation is that, in practice, CMAX exhibits a coarse-to-fine convergence trend: early iterations produce large updates that rapidly capture dominant rotational motion, whereas later iterations mainly perform fine refinement with diminishing alignment improvements, as illustrated in Figure 4. However, standard implementations do not adapt computational granularity to this convergence behavior. They keep the same number of events *N* and the same IWE resolution H×W for every iteration, repeatedly paying almost identical event-domain and image-domain costs even when the expected improvement per iteration becomes small.

This mismatch between *diminishing optimization returns* and *fixed computational cost* is particularly inefficient on edge platforms. Therefore, an explicit edge-oriented decomposition into event-domain and image-domain bottlenecks is crucial, and it motivates our coarse-to-fine strategy in Section 3, which allocates cheaper computation to early iterations and progressively increases fidelity only when fine refinement is necessary.

## 3. Coarse-to-Fine Contrast Maximization (CCMAX)

Although CMAX typically converges in a coarse-to-fine manner, standard implementations apply fixed event and image resolutions across all iterations. This mismatch between diminishing optimization gains and constant per-iteration cost calls for an adaptive, coarse-to-fine optimization strategy. Accordingly, we propose CCMAX for rotational ego-motion estimation. The key idea is to *adapt the computational granularity across optimization iterations* so that inexpensive, low-fidelity computations are used when coarse alignment is sufficient, and high-fidelity computations are reserved for the final refinement. Concretely, CCMAX combines two complementary mechanisms:

- **Coarse-to-Fine IWE Construction** progressively increases the IWE grid resolution across optimization stages, directly reducing the image-domain cost in early stages.
- **Coarse-Grid Event Subsampling** reduces redundant event contributions at coarse resolutions by selecting representative events within coarse spatial bins, reducing the event-domain cost during coarse stages.

For a given event window $E_{m}$, CCMAX runs a fixed number of optimization *stages* $\left\{1,\ldots ,k_{\max}\right\}$. Each stage *k* uses (i) a resolution scale $s_{k}\in \left(0,1\right]$ for IWE construction and (ii) a keep ratio $\rho_{k}\in \left(0,1\right]$ for event subsampling (with $\rho_{k}=1$ meaning no subsampling). Compared to the baseline cost $k_{\max}\left(O(N)+O(HW)\right)$, the stage-wise cost in CCMAX becomes approximately

(11) $$O\left(\rho_{k}N\right)+O\left(s_{k}^{2}HW\right),$$

highlighting how resolution scaling and subsampling jointly reduce the dominant costs identified in Section 2.2.

### 3.1. Coarse-to-Fine IWE Construction for Reducing Image-Domain Cost

Coarse-to-fine IWE construction reformulates the CMAX optimization on an event window $E_{m}$ as a sequence of stages with progressively increasing spatial resolution. In this context, a *stage* corresponds to a single iteration of the CMAX optimization loop: given the current motion estimate ω, events are warped and accumulated into an IWE, followed by contrast and gradient evaluation to update ω once. In CCMAX, early stages operate on downscaled IWE grids (e.g., s∈{1/4,1/2}) to form a coarse alignment at low computational cost, while later stages progressively increase the resolution (s→1) to recover fine alignment at the original grid resolution.

**Figure 5:**
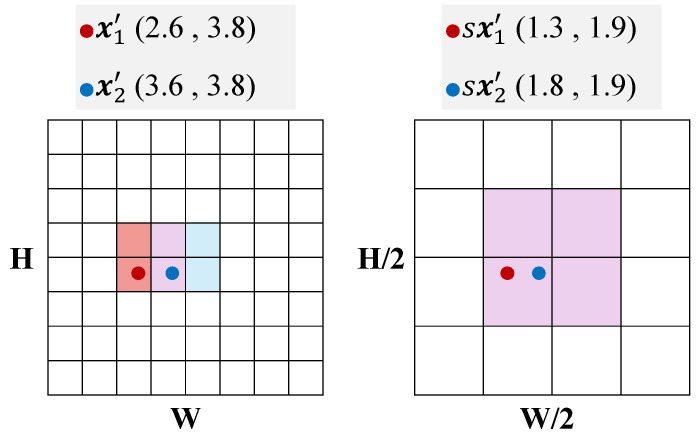
Illustration of coarse-to-fine IWE construction with resolution scale s=1/2. Warped events that would distribute over a 2×2 neighborhood on the full-resolution grid are accumulated into the same coarse grid cell via bilinear voting, reducing the effective IWE resolution while preserving dominant event alignment cues.

Starting from the IWE definition in (5), we define a scaled IWE on a grid with resolution scale $s\in \{1,\frac{1}{2},\frac{1}{4}\}$ as

(12) $$I_{s}\left(u;\omega \right)=\sum_{k=1}^{N}p_{k}\;K\;\left(u-s\;x_{k}^{\prime }\left(\omega \right)\right),\;s\in \left\{1,\frac{1}{2},\frac{1}{4}\right\},$$

where u denotes coordinates on the scaled grid and K(·) is the discrete accumulation kernel (e.g., bilinear voting). The term $s\;x_{k}^{\prime }\left(\omega \right)$ maps warped event locations into the scaled coordinate system, so that the same event set is accumulated into a smaller grid. If the original IWE resolution is H×W, the scaled grid covers $\Omega_{s}$ with resolution $H_{s}\times W_{s}$, where $H_{s}=\left\lceil sH\right\rceil$ and $W_{s}=\left\lceil sW\right\rceil$. This effect is illustrated in Figure 5 for the case s=1/2, where events that would spread over a 2×2 neighborhood on the full-resolution grid are merged into a single coarse cell.

From an edge-oriented perspective, this resolution scaling directly reduces the dominant image-domain cost. Image-domain operations in CMAX—including Gaussian smoothing, contrast (variance) computation, and gradient-related image processing—scale linearly with the number of grid cells. Since $H_{s}W_{s}\approx s^{2}HW$, reducing the resolution by a factor *s* lowers the image-domain cost by approximately $s^{2}$: for s=1/2, the cost becomes about one quarter of the full-resolution cost, and for s=1/4 it becomes about one sixteenth. This behavior directly targets the dominant O(HW) image-domain bottleneck.

Importantly, coarse IWE grids remain effective in early optimization stages. Using a coarse grid implicitly performs spatial pooling, whereby multiple nearby warped events contribute to the same grid cell. This pooling suppresses fine spatial details that are not yet necessary during early optimization, while retaining the dominant alignment structure induced by the primary rotational motion. As a result, coarse stages can provide stable and informative ascent directions at substantially reduced cost, and later stages recover precision by progressively increasing the IWE resolution.

### 3.2. Coarse-Grid Event Subsampling for Reducing Event-Domain Cost

While coarse-to-fine IWE construction reduces the image-domain cost by shrinking the IWE grid, coarse-grid event subsampling further reduces the event-domain cost by decreasing the number of events processed during coarse optimization stages. The key observation is that, at low IWE resolutions, many events become redundant because they fall into the same coarse grid cell and contribute similarly to the contrast objective. For example, when using s=1/2 in (12), events that would originally spread across a 2×2 neighborhood on the full-resolution grid are merged into a single coarse cell. As a result, contrast becomes primarily influenced by the aggregated cell-level event mass rather than by fine sub-pixel differences. This redundancy can be exploited by selecting a subset of representative events per coarse cell, preserving coarse alignment capability while reducing event processing cost [37,38].

To control the degree of subsampling, we design the keep ratio $\rho_{s}$ as a function of the resolution scale *s*. At resolution scale *s*, the number of coarse grid cells scales approximately as $s^{2}$ relative to the full-resolution grid. A naive choice $\rho_{s}=s^{2}$ would maintain the same *average* number of events per cell. However, event streams are typically highly non-uniform and temporally bursty, and overly aggressive reduction can eliminate informative alignment cues in important regions. We therefore choose

(13) $$\rho_{s}=s\;(\mathrm{yielding}\;\;\rho_{1/2}=1/2,\;\rho_{1/4}=1/4).$$

which intentionally increases the per-cell event density by a factor of 1/s compared to the full-resolution average. For instance, at s=1/2, the grid area is reduced to 1/4 while the number of processed events is reduced to 1/2, effectively doubling the average number of events per coarse cell. This design improves the robustness of coarse-stage contrast estimation while still providing substantial event-domain savings.

The subsampling procedure is summarized in Algorithm 1. For each event window, we first apply a *reference warp* using $\omega_{\mathrm{ref}}$, which we set to the warm-start estimate (e.g., from the previous window). This reference alignment increases the likelihood that events originating from the same physical edge cluster into the same coarse grid cell, making cell-wise redundancy more explicit. Each warped event is then mapped to the scaled coordinates $\left(u_{i},v_{i}\right)=s\left(x_{i}^{\prime },y_{i}^{\prime }\right)$ and assigned to a one-dimensional cell index $g_{i}=\left\lfloor v_{i}\right\rfloor W_{s}+\left\lfloor u_{i}\right\rfloor$ over the scaled domain $\Omega_{s}$.
**Algorithm 1:** Coarse-grid event subsampling
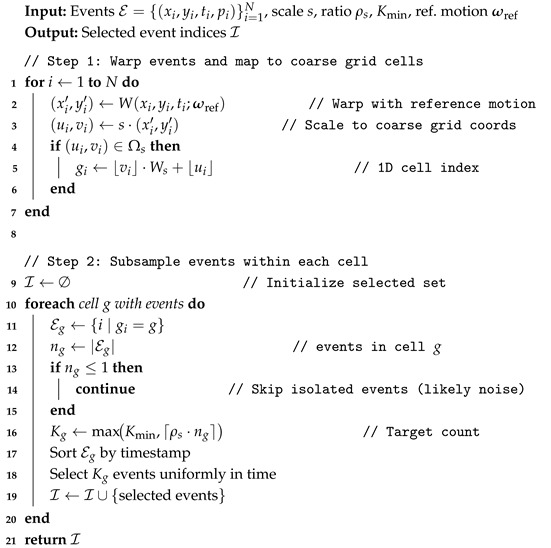


Within each coarse cell, we select $K_{g}$ events, where $K_{g}$ is proportional to the number of events in the cell via $\rho_{s}$ and is lower-bounded by $K_{\min}$. Cells containing only a single event ($n_{g}\leq 1$) are discarded, as they are more likely to correspond to isolated noise events rather than stable edge structures. Moreover, because event generation can be temporally bursty, selecting events purely at random or only from the beginning of a cell’s event list can bias the temporal distribution and distort the motion cue within the window. To mitigate this effect, we employ *time-stratified* selection: after sorting events in each cell by timestamp, we sample events approximately uniformly over the cell’s temporal extent (Algorithm 1, Lines 16–17). In CCMAX, event subsampling is applied only during coarse stages (s<1), while the final fine stage uses all events (ρ=1) to recover full-resolution accuracy.

Overall, coarse-grid event subsampling complements coarse-to-fine IWE construction. The former reduces the dominant O(N) event-domain cost by limiting the number of processed events in coarse stages, while the latter reduces the dominant O(HW) image-domain cost by shrinking the IWE grid. Together, these two mechanisms form an edge-oriented coarse-to-fine CMAX pipeline whose computational fidelity is aligned with the refinement requirements of each optimization stage.

### 3.3. Evaluation of CCMAX Configurations

Section 3.1 and Section 3.2 presented two complementary mechanisms for aligning computational fidelity with the coarse-to-fine convergence behavior of contrast maximization: (i) resolution scheduling for IWE construction (image-domain cost control) and (ii) coarse-grid event subsampling (event-domain cost control). In this subsection, we empirically evaluate how different CCMAX configurations affect estimation accuracy. The goal is twofold: first, to validate the design choices in CCMAX from an optimization perspective, and second, to understand which coarse-to-fine schedules preserve baseline-level accuracy while remaining suitable for resource-constrained edge deployment.

**Evaluation metric and setup.**

We evaluate CCMAX on the *Boxes rotation* and *Poster rotation* sequences from the Event Camera Dataset [34], which were recorded using a DAVIS event camera with a spatial resolution of 240×180 pixels. These sequences predominantly contain rotational motion with increasing angular speed and provide synchronized IMU measurements used as ground-truth angular velocity. To assess performance under different motion regimes, we analyze two disjoint temporal segments per sequence: 0–15 s (*Front*, slow motion) and 45–60 s (*Back*, fast motion). These segments are intentionally selected to contrast low- and high-angular-speed regimes, enabling a controlled analysis of the accuracy–efficiency trade-off under edge-oriented computational constraints.

The event stream is processed using overlapping event windows. Each window contains $N_{w}$ = 40,000 events, and consecutive windows are shifted by $N_{\mathrm{shift}}$ = 20,000 events (50% overlap), a common strategy to reduce boundary effects in windowed time-series analysis [39]. This window size provides sufficient event density for a stable IWE-based contrast and gradient estimation, while keeping the locally constant angular-velocity assumption reasonable within a single window. The angular velocity of the first window is initialized to zero and is not obtained from IMU measurements. The same initialization strategy is consistently applied to both the baseline and all proposed configurations. For each window *m*, the optimizer is warm-started using the previous estimate ${\hat{\omega }}_{m-1}$.

Accuracy is measured using the per-window angular-velocity error with respect to the IMU reference. Let ${\hat{\omega }}_{m}\in R^{3}$ denote the estimated angular velocity and $\omega_{m}^{\mathrm{IMU}}$ the ground truth. We define the error vector $e_{m}={\hat{\omega }}_{m}-\omega_{m}^{\mathrm{IMU}}$ and the corresponding scalar error $\varepsilon_{m}=∥e_{m}{∥}_{1}=|e_{m,x}\left|+\right|e_{m,y}\left|+\right|e_{m,z}|$. Because event streams can be bursty and occasional window-level failures may occur, we summarize the error distribution using robust statistics. Specifically, we define the ***accuracy score*** $S_{\mathrm{acc}}$ as the sum of the median and the interquartile range (IQR) of the window-level errors $\left\{\varepsilon_{m}\right\}$, where the IQR is defined as the difference between the 75th and 25th percentiles ($Q_{75}$ and $Q_{25}$):

(14) $$S_{\mathrm{acc}}=\mathrm{median}\left(\varepsilon \right)+\mathrm{IQR}\left(\varepsilon \right),\;\mathrm{IQR}\left(\varepsilon \right)=Q_{75}\left(\varepsilon \right)-Q_{25}\left(\varepsilon \right),$$

which captures both the typical error magnitude and its variability across windows. The resulting score has the same physical unit as the angular velocity (i.e., deg/s in our experiments), with lower values indicating better estimation accuracy.

**Iteration budget and configuration notation.**

**Figure 6:**
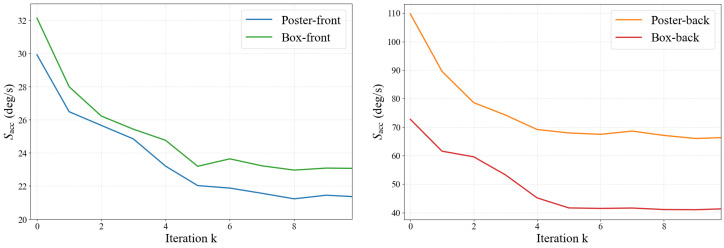
Accuracy score $S_{\mathrm{acc}}$ as a function of the iteration budget *k* for the full-resolution baseline configuration. Results are shown for slow-motion segments (Boxes-front and Poster-front, 0–15 s) and fast-motion segments (Boxes-back and Poster-back, 45–60 s).

Contrast maximization is inherently iterative, and unconstrained iteration counts can lead to unpredictable runtime. Since our target setting is edge deployment, where bounded latency is desirable, we adopt a fixed maximum iteration budget. Figure 6 reports $S_{\mathrm{acc}}$ of a full-resolution baseline configuration as a function of the iteration budget *k*. Across all four scenarios, the accuracy improves rapidly during the first few iterations and exhibits diminishing returns thereafter, with only marginal improvement observed for k≥5. Accordingly, we set $k_{\max}=5$ for all subsequent experiments and treat each iteration as one stage.

A CCMAX configuration is represented by a length-$k_{\max}$ stage string, where each stage specifies the IWE resolution scale *s* and, when enabled, the event keep ratio $\rho_{s}$. We consider three stage types: F (*fine*), with full-resolution IWE construction (s=1) and no event subsampling (ρ=1); C1 (*coarse-1*), with half-resolution IWEs ($s=\frac{1}{2}$); and C2 (*coarse-2*), with quarter-resolution IWEs ($s=\frac{1}{4}$). When event subsampling is enabled, coarse stages follow the rule $\rho_{s}=s$, while fine stages always use all events (ρ=1). For example, the configuration C2C2C1C1F corresponds to a five-stage schedule in which stages 1–2 use $s=\frac{1}{4}$ and $\rho =\frac{1}{4}$, stages 3–4 use $s=\frac{1}{2}$ and $\rho =\frac{1}{2}$, and the final stage uses full resolution with s=1 and ρ=1. The full-resolution baseline used for comparison corresponds to FFFFF, where all stages operate at full IWE resolution using all events.

For experiments that isolate the effect of coarse-to-fine IWE construction, event subsampling is disabled and all stages use the full event set (i.e., $\rho_{s}=1$ for all *s*), so C1 and C2 denote resolution scaling only. For experiments that evaluate coarse-grid event subsampling, subsampling is applied only in coarse stages following the scheme described in Section 3.2.

**Effect of coarse-to-fine IWE construction.**

**Figure 7:**
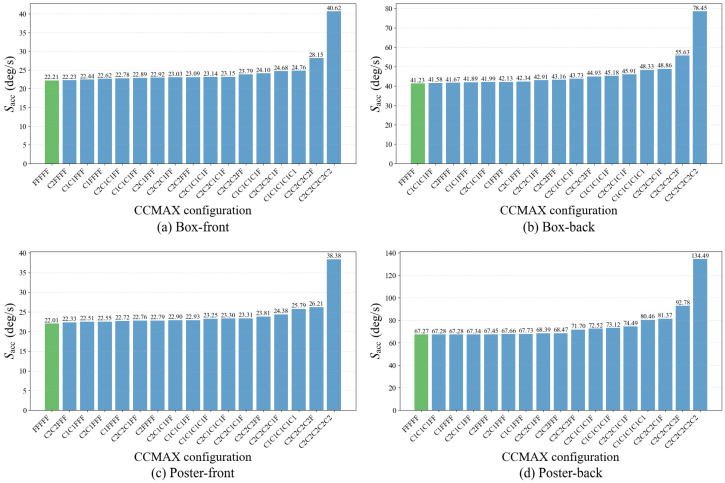
Effect of coarse-to-fine IWE construction without event subsampling. The accuracy score $S_{\mathrm{acc}}$ is reported for different stage-resolution schedules and evaluated against IMU ground truth. Each subfigure corresponds to a different scenario, as labeled in the figure. The full-resolution baseline corresponds to the FFFFF configuration. Lower values of $S_{\mathrm{acc}}$ indicate better estimation accuracy.

Figure 7 compares $S_{\mathrm{acc}}$ across different stage-resolution schedules when event subsampling is disabled. As expected, *coarse-only* schedules, such as C2C2C2C2C2 and C1C1C1C1C1, consistently lead to substantial accuracy degradation across all scenarios. Without any fine stage, the optimization lacks high-resolution spatial cues required for final refinement, and residual misalignment remains. This effect is particularly pronounced in challenging scenarios such as Poster-back, where the baseline score of 67.27 increases to 134.49 under the C2C2C2C2C2 configuration.

In contrast, *mixed* coarse-to-fine schedules that include one or more fine stages are able to recover most of the baseline accuracy. This behavior reflects the intended role of resolution scheduling. Early coarse stages perform a low-pass aggregation of warped events: nearby events are merged into shared coarse cells, preserving dominant alignment trends while suppressing fine spatial details. As a result, these stages are effective for rapidly capturing the dominant motion components at reduced image-domain cost. Subsequent fine stages reintroduce high-frequency spatial structure in the IWE, enabling precise correction of the residual error left by the coarse alignment.

This effect can be clearly observed when a fine stage is appended to an otherwise coarse-only schedule. For example, switching from C2C2C2C2C2 to C2C2C2C2F reduces the accuracy score from 40.62 to 28.15 in the Box-front scenario, and from 134.49 to 92.78 in the Poster-back scenario, demonstrating the strong corrective effect of even a single fine refinement stage. Configurations that combine intermediate-resolution and fine stages, such as C2C2C1FF and C2C1C1C1F, further stabilize performance, yielding scores of 68.39 and 72.52 in Poster-back, respectively.

From a cost perspective, these results also highlight an important trade-off. Because image-domain cost scales linearly with the IWE resolution, placing too many fine stages significantly increases the cumulative computation over all iterations. Nevertheless, configurations with a single fine stage already achieve a substantial recovery relative to coarse-only schedules, indicating that most of the benefit of high-resolution refinement can be obtained with limited fine-stage usage. Overall, these results justify coarse-to-fine IWE construction as an edge-oriented design: image-domain cost is reduced through low-resolution early stages, while accuracy degradation is effectively mitigated by reserving a small number of fine stages. The stage configuration can be adapted to different accuracy requirements and computational budgets.

**Effect of coarse-grid event subsampling.**

**Figure 8:**
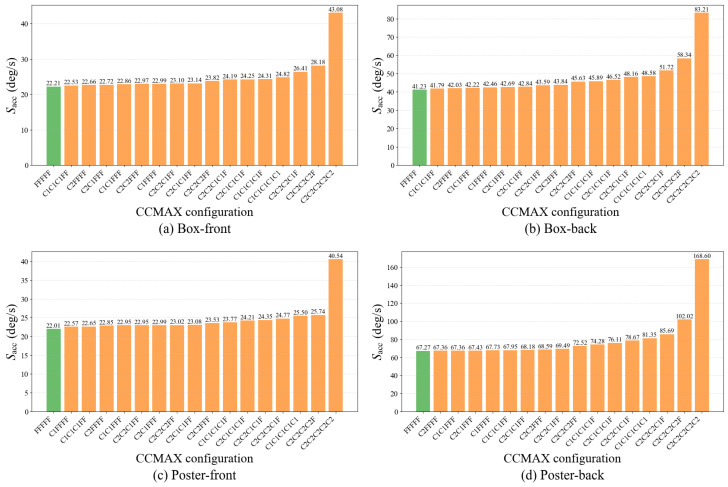
Effect of coarse-grid event subsampling. $S_{\mathrm{acc}}$ is reported for different coarse-to-fine schedules with subsampling applied in coarse stages, while fine stages always use all events, and evaluated against IMU ground truth. Each subfigure corresponds to a different scenario, as labeled in the figure. The full-resolution baseline corresponds to the FFFFF configuration. Lower values of $S_{\mathrm{acc}}$ (deg/s) indicate better estimation accuracy.

Figure 8 evaluates the effect of adding coarse-grid event subsampling on top of the same coarse-to-fine resolution schedules. In these experiments, subsampling is applied only in coarse stages, while fine stages always use the full event set. Across mixed coarse-to-fine schedules that include at least one fine stage, introducing event subsampling results in only minor changes in $S_{\mathrm{acc}}$, indicating that a substantial reduction in the number of processed events can be achieved without materially degrading estimation accuracy.

For example, in the C2C2C1C1F configuration, event subsampling reduces the number of events by approximately one quarter in the C2 stages and one half in the C1 stages. Despite this aggressive reduction, the increase in $S_{\mathrm{acc}}$ remains limited, ranging from 1.04 to 4.18 across the four scenarios. Similarly, in C1C1C1C1F, the difference introduced by subsampling ranges from 0.21 to 1.71, and becomes even smaller (0.21 to 0.67) when two fine stages are included, as in C1C1C1FF. These results show that coarse-grid event subsampling has a consistently limited impact on accuracy when sufficient fine-stage refinement is preserved.

This robustness can be explained by the redundancy structure exploited by the subsampling scheme. With warm-start initialization, events are already partially aligned under the reference motion, so events generated by the same physical edge tend to cluster into the same coarse grid cell after warping and scaling. At low resolutions, the contrast objective and its gradient are driven primarily by aggregated cell-level contributions rather than by the exact sub-pixel placement of individual events. As a result, many events within the same cell are redundant for coarse-stage optimization. By retaining a fixed fraction of events per active cell and enforcing time-stratified sampling, the proposed subsampling scheme preserves both spatial and temporal coverage of motion cues within each window.

Importantly, when at least one fine stage is included, most configurations maintain consistent accuracy regardless of whether subsampling is applied. In other words, the presence of a fine stage is sufficient to compensate for the information loss introduced by event reduction in coarse stages, allowing subsampling to be used aggressively without degrading final estimation accuracy.

In contrast, the limitations of subsampling become apparent in configurations that lack fine refinement. When all stages operate at the lowest resolution, as in C2C2C2C2C2, adding subsampling further amplifies the accuracy degradation, with the accuracy score increasing by a factor of 1.84–2.51 relative to the baseline across the four scenarios. This behavior reflects the fundamental limitation of coarse-only optimization: without any high-resolution stage, neither resolution scheduling nor subsampling can recover the fine alignment details required for accurate estimation.

The primary contribution of coarse-grid event subsampling lies in reducing the event-domain cost. Because event warping and bilinear voting are performed on a per-event basis, the event-domain cost scales linearly with the number of processed events. For example, in the C2C2C1C1F configuration, reducing the number of events to approximately one quarter in the first two stages results in an event-domain cost reduction of about 75% in those stages, and retaining approximately one-half of the events in the C1 stages yields a corresponding event-domain cost reduction of about 50%. This reduction complements the image-domain cost savings achieved by coarse-to-fine IWE construction.

In summary, the proposed CCMAX framework achieves substantial computational savings in both the image and event domains while maintaining nearly the same estimation accuracy. This balance between robust error performance and computational efficiency justifies CCMAX as an edge-oriented design that explicitly accounts for both accuracy and resource constraints.

## 4. Experimental Evaluation

In this section, we quantify the efficiency benefits of CCMAX from two complementary perspectives. First, we provide a platform-agnostic compute analysis based on FLOPs and characterize the accuracy–efficiency trade-off across the configuration design space. Second, we verify that these computational savings translate into tangible energy reductions on a prototype edge SoC platform.

### 4.1. Compute Analysis for Edge Deployment

$S_{\mathrm{acc}}$ in (14) provides a robust absolute error measure for each evaluation segment. However, because the four segments considered in Section 3.3 exhibit substantially different motion magnitudes, directly comparing absolute errors across scenarios can be misleading. To enable a compact, motion-scale-agnostic comparison, we additionally introduce a normalized summary metric referred to as *Average % of Peak Motion*.

Let $\omega_{j}^{\mathrm{peak}}$ denote the peak ground-truth angular velocity for scenario *j*. For the four evaluation segments, these values are 300 deg/s (Boxes-front), 700 deg/s (Boxes-back), 300 deg/s (Poster-front), and 1000 deg/s (Poster-back). We define the scenario-normalized score as

(15) $$\eta_{j}=100\cdot \frac{S_{\mathrm{acc},j}}{\omega_{j}^{\mathrm{peak}}}\;\left[\%\right],$$

and summarize performance using the average $\bar{\eta }=\frac{1}{4}\sum_{j=1}^{4}\eta_{j}$. This metric preserves the robustness of $S_{\mathrm{acc}}$ while enabling direct comparison across scenarios with different motion scales.

**Figure 9:**
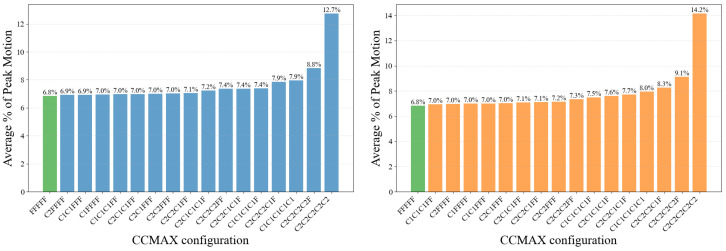
Average % of Peak Motion across the four evaluation scenarios for all CCMAX configurations. Results are averaged over Boxes-front, Boxes-back, Poster-front, and Poster-back. Left: configurations without coarse-grid event subsampling. Right: configurations with coarse-grid event subsampling applied in coarse stages. Lower values indicate better motion-normalized estimation accuracy.

Figure 9 reports the average normalized error $\bar{\eta }$ for all configurations. For the full-resolution baseline FFFFF, the average normalized error is 6.8% across the four scenarios. Because the CMAX optimization process is iterative and not strictly deterministic, we observe small but consistent relative variations in this normalized error even for the same configuration. Based on empirical evaluation of the baseline, this variation remains within approximately 1 percentage point, which we adopt as a conservative tolerance for acceptable accuracy degradation in edge deployment.

Using this tolerance, configurations that include one fine stage are partially edge-feasible, and those with two or more fine stages are consistently within the acceptable accuracy range, whereas coarse-only schedules fall outside the tolerance. Within the tolerance region, increasing the number of fine stages leads to only marginal accuracy improvement, while the computational overhead of the CMAX pipeline grows rapidly due to repeated full-resolution image-domain processing. As a result, configurations with exactly one fine stage form the most compact accuracy–efficiency trade-off near the tolerance boundary, whereas additional fine stages yield diminishing accuracy gains relative to their cost increase. Accordingly, the subsequent analysis focuses on configurations with a single fine stage, which best capture the practical trade-off between estimation accuracy and computational efficiency.

To quantify computational cost in a platform-agnostic manner, we measure FLOPs following common practice in efficiency analysis [40,41]. Additions and multiplications are counted explicitly, and constant divisions are replaced by multiplications using precomputed reciprocals so that all configurations are evaluated under the same counting rules. As discussed in Section 2.2, the dominant cost of contrast maximization arises from repeated execution of the CMAX pipeline. This cost is governed by two factors: the number of processed events, which determines the event-domain cost of warping and bilinear voting, and the spatial resolution of the IWE, which determines the image-domain cost of smoothing and contrast/gradient evaluation. Accordingly, reducing the event count through coarse-grid subsampling and reducing the IWE resolution through coarse-to-fine scheduling directly target the two dominant contributors to the overall FLOPs. Finally, we verified that the arithmetic overhead of the iterative optimizer itself is negligible compared to the pipeline cost; in our measurements, optimizer-related FLOPs account for less than $10^{-6}$ of the total, confirming that the FLOPs reduction is attributable to changes in the CMAX pipeline itself, with optimizer overhead being negligible.

**Figure 10:**
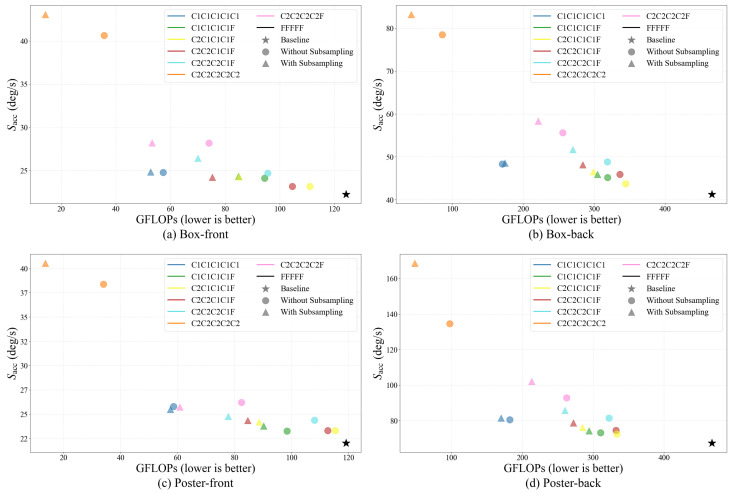
Design-space characterization on the FLOPs–accuracy plane for CCMAX configurations.

Figure 10 visualizes the design space in terms of total FLOPs and the accuracy score $S_{\mathrm{acc}}$. Two consistent trends emerge across all scenarios. First, enabling coarse-grid event subsampling systematically shifts configurations toward lower FLOPs, confirming that reducing the number of processed events in coarse stages directly lowers the event-domain cost. Second, coarse-only schedules achieve very large FLOPs reductions but incur unacceptable accuracy degradation. For instance, C2C2C2C2C2 achieves approximately 89% and 91% FLOPs reduction on Boxes-front and Boxes-back, respectively, but degrades the accuracy score to roughly 1.9–2.0× the baseline, indicating limited practical utility. Similarly, C1C1C1C1C1 reduces FLOPs by about 58% (Boxes-front) and 63% (Boxes-back), yet still exhibits clear accuracy loss and is not edge-feasible under the baseline-accuracy criterion.

Among mixed schedules that include a fine stage, the accuracy–efficiency trade-off becomes substantially more favorable, but it remains strongly configuration dependent. In particular, introducing a single fine stage is sufficient to recover most of the baseline accuracy, whereas adding additional fine stages yields diminishing accuracy gains while rapidly increasing FLOPs due to the dominance of full-resolution image-domain computation. Using the motion-normalized tolerance defined in Figure 9, this trend becomes quantitative.

Across the four scenarios, C2C2C2C2F achieves a large FLOPs reduction of 53.4% relative to FFFFF, but exceeds the acceptable accuracy margin by +2.3 p.p., indicating that aggressive early coarsening without sufficient refinement is insufficient. In contrast, C1C1C1C1F remains within the tolerance (+0.7 p.p.) with a moderate FLOPs reduction of 34.3%, while C2C2C1C1F provides a stronger reduction of 39.2% FLOPs while still satisfying the accuracy constraint (+0.9 p.p.). These results highlight that the resolution level and duration of the early coarse stages play a critical role in determining the final accuracy–efficiency balance, even when the number of fine stages is fixed. Notably, C2C2C1C1F exhibits consistent FLOPs reductions across all scenarios (approximately 39% on Boxes-front, 39% on Boxes-back, 29% on Poster-front, and 42% on Poster-back), indicating robust efficiency gains over a wide range of motion regimes.

Overall, the FLOPs analysis supports the design rationale of CCMAX: allocating coarse computation to early iterations and reserving a limited fine refinement stage enables a favorable accuracy–efficiency trade-off under resource-constrained edge settings.

### 4.2. Energy Validation on a Prototype Edge SoC

**Figure 11:**
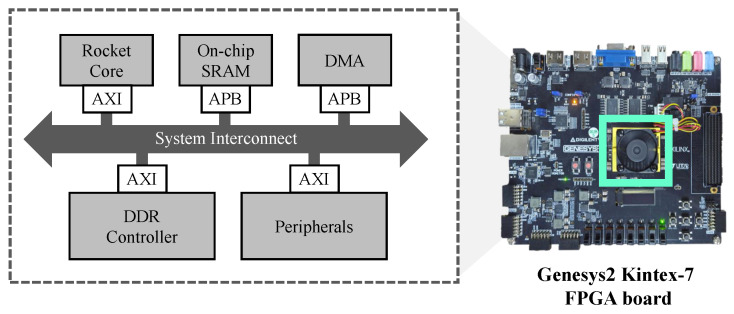
Prototype edge SoC platform implemented on a Genesys2 (Kintex-7) FPGA board. The platform integrates a 50 MHz RISC-V Rocket core, on-chip SRAM, a DDR controller, and μNoC, and is used to evaluate the energy consumption of the proposed CCMAX pipeline.

To directly validate that the computational savings achieved by CCMAX translate into tangible energy reductions in edge settings, we measure the energy consumption of the proposed method on a prototype edge-class SoC platform. Figure 11 illustrates the overall architecture of the prototype system.

The prototype processor is designed based on RISC-V eXpress (RVX), an EDA tool widely adopted for developing edge processors on the RISC-V platform [8,42,43,44,45], and integrates a low-power Rocket core [46] operating at 50 MHz. As shown in Figure 11, this platform consists of 128 KB of on-chip SRAM, a DDR controller, and a low-power μNoC [47] with AXI/APB interconnect. The design is validated on a Genesys2 Kintex-7 FPGA board [48], and power estimation is performed using a 45 nm technology model [49]. FPGA resource utilization and power consumption breakdown are summarized in Table 1. Resource usage is reported based on Xilinx Vivado [50] synthesis results, and power analysis for the same RTL configuration is carried out using Synopsys Design Compiler [51], which reports a total system power of 57.01 mW.

To quantify the energy benefit of CCMAX at the platform level, we focus on the dominant computational bottleneck of the algorithm: the repeated CMAX pipeline shown in Figure 3, which consists of event warping, IWE accumulation, and contrast/gradient evaluation. This pipeline block concentrates both arithmetic operations and memory accesses, and it is executed identically at every optimization stage. As confirmed in the FLOPs analysis, this block dominates the overall computation and energy budget, while the overhead of the iterative optimizer itself is negligible. Accordingly, energy measurements are targeted specifically at this pipeline to directly capture the impact of reduced event-domain and image-domain workloads. This targeted measurement does not aim to capture the end-to-end system energy consumption of a complete SLAM pipeline. Instead, it is intended to evaluate the transferability of algorithmic computational savings—introduced by coarse-to-fine scheduling and event reduction—to hardware-level energy consumption in a controlled and repeatable manner.

Energy measurements are conducted under the same fixed-size event-window processing setup used in Section 3.3. For the Fine baseline configuration, each window processes $N_{\mathrm{target}}$ = 40,000 events and uses the full IWE resolution of 180×240. To examine how energy scales with reduced event count and reduced IWE resolution, we measure two representative coarse configurations. Configuration C1 uses a resolution scale of $s=\frac{1}{2}$, corresponding to a 90×120 IWE, and processes an average of approximately 20,800 events per window. Configuration C2 uses $s=\frac{1}{4}$, corresponding to a 45×60 IWE, and processes an average of approximately 10,500 events per window. These two configurations are selected because they isolate the effect of reducing the number of processed events and the IWE resolution on the dominant CMAX pipeline, enabling direct quantification of event-domain and image-domain energy scaling at the platform level.

Table 2 reports the measured energy consumption of the CMAX pipeline normalized to the Fine baseline (Fine = 100%). The measured normalized energies are 35.47% for C1 and 12.97% for C2, which correspond to energy reductions of approximately 2.82× and 7.71×, respectively. These results demonstrate that coarse-to-fine configuration alone, without any change to the underlying hardware, can yield substantial energy savings on an edge-class SoC.

If pipeline energy were dominated solely by event-domain processing, reducing the number of events by half would be expected to yield approximately 50% of the baseline energy consumption. However, C1 consumes only 35.47% of the baseline energy, indicating that image-domain savings due to the quadratic reduction in IWE pixel count also play a significant role. To further analyze this behavior, we consider a simple two-term energy model:

(16) $$E\approx \alpha \rho +\left(1-\alpha \right)s^{2},$$

where ρ and $s^{2}$ denote the event-count and pixel-count ratios relative to the Fine baseline. Fitting this model to the C1 measurement yields an estimated image-domain energy contribution of approximately 1−α≈0.58 under the baseline setting. Using the same model to predict the energy of C2 gives an expected normalized energy of approximately 14.1%, which closely matches the measured value of 12.97% (within ∼1.1 p.p.).

Overall, these measurements corroborate the edge-oriented cost decomposition discussed earlier. The energy consumption of the CMAX pipeline is jointly governed by event-domain and image-domain costs, and reducing only one of these components is insufficient for maximal efficiency. CCMAX is explicitly designed to reduce both terms: coarse-grid event subsampling directly lowers the event-domain cost by reducing the number of processed events, while coarse-to-fine IWE construction structurally reduces the image-domain cost by shrinking the IWE resolution. The observed energy reductions confirm that the FLOPs savings achieved by CCMAX can be transferred into tangible hardware-level energy benefits on a representative edge SoC platform, supporting CCMAX as a feasible and energy-efficient motion-estimation front-end for resource-constrained edge systems.

## 5. Discussion

In this work, we focus on rotational ego-motion to enable a controlled analysis of the computational efficiency of contrast maximization (CMAX). Pure rotation admits a depth-independent warping model, which allows us to isolate how the dominant computational components of CMAX—namely, event-domain processing and image-domain IWE construction—scale under different coarse-to-fine configurations, without confounding effects from scene geometry or depth estimation.

Extending the same approach to motion models that include translation introduces additional challenges. With translation, event warping becomes depth-dependent and different scene points experience different apparent motions due to parallax. In practice, this typically requires additional scene representation (e.g., depth/inverse-depth, planar modeling, or a map) or joint optimization over pose and structure, which increases the number of unknowns and the per-iteration complexity of event warping, IWE construction, and contrast/gradient evaluation. These factors can further exacerbate runtime and energy constraints on resource- and power-limited edge platforms. Designing depth-aware coarse-to-fine schedules that retain informative parallax cues while maintaining computational efficiency remains an important direction for future work.

Beyond modeling considerations, event-based motion estimation is influenced by non-ideal real-world conditions. Sensor jitter, high-frequency vibration, mechanical shocks, independently moving objects, and loss of fine visual details can alter the spatiotemporal distribution of events. Such disturbances may reduce IWE contrast, introduce outlier or inconsistent event patterns, and flatten the objective-function landscape, weakening informative gradients and degrading optimization stability [52].

In the present work, we do not explicitly model or compensate for these factors, as our primary goal is to analyze and reduce the computational and energy cost of contrast maximization under the rotational setting. CCMAX should be viewed as a computation-efficient scheduling layer that preserves the original CMAX objective, and thus can be combined with complementary robustness mechanisms such as background-activity filtering, adaptive window sizing, inertial sensing or sensor fusion, and robust/weighted objective formulations. Developing CMAX frameworks that jointly address robustness under real-world disturbances while preserving computational efficiency is essential for reliable deployment and remains an open research challenge.

## 6. Conclusions

In conclusion, the main contributions, findings, and limitations of this work are summarized as follows:

1. We analyzed the computational inefficiency of contrast maximization (CMAX) for iterative rotational ego-motion estimation and identified two dominant cost components: event-domain processing that scales with the number of events, and image-domain IWE processing that scales with IWE resolution.
2. We proposed coarse-to-fine contrast maximization (CCMAX), which aligns computational fidelity with the coarse-to-fine convergence behavior of CMAX via (i) coarse-to-fine IWE construction and (ii) coarse-grid event subsampling, while explicitly retaining a final full-resolution refinement stage.
3. Experiments on standard event-camera benchmarks with IMU ground truth show that properly designed schedules achieve accuracy comparable to the full-resolution baseline under a fixed iteration budget.
4. CCMAX reduces floating-point operations by up to 42% and achieves up to 87% lower energy consumption for the iterative CMAX pipeline on a custom RISC-V–based edge SoC prototype, demonstrating suitability for real-time edge SLAM front-end deployment under tight compute and power constraints.
5. Limitations and future work: The proposed approach focuses on rotational ego-motion under a depth-independent warping model. Extensions to translation-including motion, which introduce depth dependence and parallax, as well as improved robustness to non-ideal real-world conditions (e.g., jitter, shocks, and dynamic objects), remain important directions for future research.

## Figures and Tables

**Table 1:** FPGA resource utilization and power consumption breakdown of the prototype edge SoC platform. Resource usage is reported based on Xilinx Vivado synthesis, and power is estimated using Synopsys Design Compiler under a 45 nm technology model.

IPs	LUTs	FFs	$P_{\mathrm{total}}$ (mW)
Developed Processor	32,092	27,563	57.01
⌞ RISC-V Rocket Core	14,862	10,042	6.77
⌞ Rocket Core Interface	165	201	0.62
⌞ External I/O	3108	2479	5.62
⌞ Main Memory	183	328	1.24
⌞ System Interconnect	4754	6571	6.37
⌞ DDR Controller	8043	7434	34.5
⌞ DMA	977	508	1.89

**Table 2:** Prototype edge SoC energy measurement of the CMAX pipeline (normalized to Fine).

Config.	$s_{k}^{2}$ (IWE Pixels)	$\rho_{k}$ (Events)	Norm. Energy
Fine (baseline)	1	1	100.0%
C1	1/4	1/2	35.47%
C2	1/16	1/4	12.97%

## Data Availability

The datasets analyzed in this study are publicly available. All original contributions presented in this article are included in the manuscript. Further inquiries can be directed to the corresponding author.

## References

[1] Cadena C., Carlone L., Carrillo H., Latif Y., Scaramuzza D., Neira J., Reid I., Leonard J.J. (2016). Past, Present, and Future of Simultaneous Localization and Mapping: Toward the Robust-Perception Age. IEEE Trans. Robot..

[2] Macario Barros A., Michel M., Moline Y., Corre G., Carrel F. (2022). A Comprehensive Survey of Visual SLAM Algorithms. Robotics.

[3] Chen L., Li G., Xie W., Tan J., Li Y., Pu J., Chen L., Gan D., Shi W. (2024). A Survey of Computer Vision Detection, Visual SLAM Algorithms, and Their Applications in Energy-Efficient Autonomous Systems. Energies.

[4] Jia G., Li X., Zhang D., Xu W., Lv H., Shi Y., Cai M. (2022). Visual-SLAM Classical Framework and Key Techniques: A Review. Sensors.

[5] Wang B., Song X., Lu K., Yang L. (2025). Research and Application of SLAM Algorithm for Mobile Robots in Indoor Dynamic Scene. Proceedings of the International Conference on Control and Intelligent Robotics.

[6] Sahili A.R., Hassan S., Sakhrieh S.M., Mounsef J., Maalouf N., Arain B., Taha T. (2023). A Survey of Visual SLAM Methods. IEEE Access.

[7] Cimarelli C., Millan-Romera J.A., Voos H., Sanchez-Lopez J.L. (2025). Hardware, Algorithms, and Applications of the Neuromorphic Vision Sensor: A Review. Sensors.

[8] Choi J., Choi E., Choi S., Lee W. (2025). E-BTS: A low-power Event-driven Blink Tracking System with hardware-software co-optimized design for real-time driver drowsiness detection. Alex. Eng. J..

[9] Rebecq H., Horstschaefer T., Gallego G., Scaramuzza D. (2017). EVO: A Geometric Approach to Event-Based 6-DOF Parallel Tracking and Mapping in Real Time. IEEE Robot. Autom. Lett..

[10] Gallego G., Scaramuzza D. (2017). Accurate Angular Velocity Estimation with an Event Camera. IEEE Robot. Autom. Lett..

[11] Gallego G., Rebecq H., Scaramuzza D. A Unifying Contrast Maximization Framework for Event Cameras, with Applications to Motion, Depth, and Optical Flow Estimation. Proceedings of the 2018 IEEE/CVF Conference on Computer Vision and Pattern Recognition.

[12] Shiba S., Klose Y., Aoki Y., Gallego G. (2024). Secrets of Event-Based Optical Flow, Depth and Ego-Motion Estimation by Contrast Maximization. IEEE Trans. Pattern Anal. Mach. Intell..

[13] Guo S., Gallego G. (2024). CMax-SLAM: Event-Based Rotational-Motion Bundle Adjustment and SLAM System Using Contrast Maximization. IEEE Trans. Robot..

[14] Yamaki R., Shiba S., Guillermo G., Aoki Y. Iterative Event-based Motion Segmentation by Variational Contrast Maximization. Proceedings of the IEEE/CVF Conference on Computer Vision and Pattern Recognition (CVPR) Workshops.

[15] Lichtsteiner P., Posch C., Delbruck T. (2008). A 128× 128 120 dB 15 *μ*s Latency Asynchronous Temporal Contrast Vision Sensor. IEEE J. Solid State Circuits.

[16] Posch C., Serrano-Gotarredona T., Linares-Barranco B., Delbruck T. (2014). Retinomorphic Event-Based Vision Sensors: Bioinspired Cameras with Spiking Output. Proc. IEEE.

[17] Gallego G., Delbrück T., Orchard G., Bartolozzi C., Taba B., Censi A., Leutenegger S., Davison A.J., Conradt J., Daniilidis K. (2022). Event-Based Vision: A Survey. IEEE Trans. Pattern Anal. Mach. Intell..

[18] Zhu A.Z., Yuan L., Chaney K., Daniilidis K. (2018). EV-FlowNet: Self-supervised optical flow estimation for event-based cameras. arXiv.

[19] Zhu A.Z., Yuan L., Chaney K., Daniilidis K. Unsupervised Event-Based Learning of Optical Flow, Depth, and Egomotion. Proceedings of the IEEE/CVF Conference on Computer Vision and Pattern Recognition (CVPR).

[20] Tian Y., Andrade-Cetto J. (2023). Egomotion from event-based SNN optical flow. Proceedings of the 2023 International Conference on Neuromorphic Systems.

[21] Qu D., Yan C., Wang D., Yin J., Chen Q., Xu D., Zhang Y., Zhao B., Li X. Implicit Event-RGBD Neural SLAM. Proceedings of the IEEE/CVF Conference on Computer Vision and Pattern Recognition (CVPR).

[22] Li W., Liao B., Zhou Y., Xu Q., Wan P., Liu P. (2025). E-MoFlow: Learning Egomotion and Optical Flow from Event Data via Implicit Regularization. arXiv.

[23] Zhu A.Z., Atanasov N., Daniilidis K. Event-Based Visual Inertial Odometry. Proceedings of the 2017 IEEE Conference on Computer Vision and Pattern Recognition (CVPR).

[24] Mueggler E., Gallego G., Rebecq H., Scaramuzza D. (2018). Continuous-Time Visual-Inertial Odometry for Event Cameras. IEEE Trans. Robot..

[25] Wang K., Zhao K., Lu W., You Z. (2025). Stereo Event-Based Visual–Inertial Odometry. Sensors.

[26] Kueng B., Mueggler E., Gallego G., Scaramuzza D. Low-latency visual odometry using event-based feature tracks. Proceedings of the 2016 IEEE/RSJ International Conference on Intelligent Robots and Systems (IROS).

[27] Yang L. (2022). Ego-motion Estimation Based on Fusion of Images and Events. arXiv.

[28] Jawaid M., Märtens M., Chin T.J. (2026). Event-RGB fusion for spacecraft pose estimation under harsh lighting. Aerosp. Sci. Technol..

[29] Kim H., Kim H.J. (2021). Real-Time Rotational Motion Estimation with Contrast Maximization Over Globally Aligned Events. IEEE Robot. Autom. Lett..

[30] Zhu A.Z., Atanasov N., Daniilidis K. Event-based feature tracking with probabilistic data association. Proceedings of the 2017 IEEE International Conference on Robotics and Automation (ICRA).

[31] Mitrokhin A., Fermüller C., Parameshwara C., Aloimonos Y. Event-Based Moving Object Detection and Tracking. Proceedings of the 2018 IEEE/RSJ International Conference on Intelligent Robots and Systems (IROS).

[32] Zhou Y., Gallego G., Lu X., Liu S., Shen S. (2023). Event-Based Motion Segmentation with Spatio-Temporal Graph Cuts. IEEE Trans. Neural Netw. Learn. Syst..

[33] Nah S., Baik S., Hong S., Moon G., Son S., Timofte R., Mu Lee K. Ntire 2019 challenge on video deblurring and super-resolution: Dataset and study. Proceedings of the IEEE/CVF Conference on Computer Vision and Pattern Recognition Workshops.

[34] Mueggler E., Rebecq H., Gallego G., Delbruck T., Scaramuzza D. (2017). The event-camera dataset and simulator: Event-based data for pose estimation, visual odometry, and SLAM. Int. J. Robot. Res..

[35] Fletcher R., Reeves C.M. (1964). Function minimization by conjugate gradients. Comput. J..

[36] Polak E., Ribière G. (1969). Note sur la convergence de méthodes de directions conjuguées. Revue Française D’Informatique et de Recherche Opérationnelle. Série Rouge.

[37] Gehrig D., Scaramuzza D. (2022). Are High-Resolution Event Cameras Really Needed?. arXiv.

[38] Araghi H., van Gemert J., Tomen N. Making Every Event Count: Balancing Data Efficiency and Accuracy in Event Camera Subsampling. Proceedings of the IEEE/CVF Conference on Computer Vision and Pattern Recognition (CVPR) Workshops.

[39] Allen J. (2003). Short term spectral analysis, synthesis, and modification by discrete Fourier transform. IEEE Trans. Acoust. Speech Signal Process..

[40] Roth W., Schindler G., Klein B., Peharz R., Tschiatschek S., Fröning H., Pernkopf F., Ghahramani Z. (2024). Resource-efficient neural networks for embedded systems. J. Mach. Learn. Res..

[41] Somvanshi S., Islam M.M., Chhetri G., Chakraborty R., Mimi M.S., Shuvo S.A., Islam K.S., Javed S., Rafat S.A., Dutta A. (2025). From Tiny Machine Learning to Tiny Deep Learning: A Survey. ACM Comput. Surv..

[42] Han K., Lee S., Oh K.I., Bae Y., Jang H., Lee J.J., Lee W., Pedram M. (2021). Developing TEI-Aware Ultralow-Power SoC Platforms for IoT End Nodes. IEEE Internet Things J..

[43] Park J., Han K., Choi E., Lee J.J., Lee K., Lee W., Pedram M. (2024). Designing Low-Power RISC-V Multicore Processors with a Shared Lightweight Floating Point Unit for IoT Endnodes. IEEE Trans. Circuits Syst. I Regul. Pap..

[44] Jeon S., Lee K., Lee K., Lee W. (2024). Dynamic Performance and Power Optimization with Heterogeneous Processing-in-Memory for AI Applications on Edge Devices. Micromachines.

[45] Lee K., Jeon S., Lee K., Lee W., Pedram M. (2025). Radar-PIM: Developing IoT Processors Utilizing Processing-in-Memory Architecture for Ultrawideband-Radar-Based Respiration Detection. IEEE Internet Things J..

[46] SiFIVE. https://github.com/chipsalliance/rocket-chip.

[47] Han K., Lee J.J., Lee J., Lee W., Pedram M. (2018). TEI-NoC: Optimizing Ultralow Power NoCs Exploiting the Temperature Effect Inversion. IEEE Trans. Comput. Aided Des. Integr. Circuits Syst..

[48] Xilinx. https://www.amd.com/en/products/adaptive-socs-and-fpgas/fpga/kintex-7.html.

[49] NCSU FreePDK45. https://eda.ncsu.edu/freepdk/freepdk45.

[50] Xilinx Vivado. https://www.xilinx.com/support/download.html.

[51] Synopsys Design Compiler. https://www.synopsys.com/implementation-and-signoff/rtl-synthesis-test/dc-ultra.html.

[52] Stoffregen T., Kleeman L. Event Cameras, Contrast Maximization and Reward Functions: An Analysis. Proceedings of the 2019 IEEE/CVF Conference on Computer Vision and Pattern Recognition (CVPR).

